# Maternal Height and Child Growth Patterns

**DOI:** 10.1016/j.jpeds.2013.02.002

**Published:** 2013-08

**Authors:** O. Yaw Addo, Aryeh D. Stein, Caroline H. Fall, Denise P. Gigante, Aravinda M. Guntupalli, Bernardo L. Horta, Christopher W. Kuzawa, Nanette Lee, Shane A. Norris, Poornima Prabhakaran, Linda M. Richter, Harshpal S. Sachdev, Reynaldo Martorell, Consortium on Health Orientated Research in Transitional Societies (COHORTS) Group

**Affiliations:** 1 Hubert Department of Global Health, Rollins School of Public Health, Emory University, Atlanta, GA; 2 Medical Research Council Lifecourse Epidemiology Unit, University of Southampton, Southampton, United Kingdom; 3 Postgraduate Program in Epidemiology, Universidade Federal de Pelotas, Pelotas, Brazil; 4 Center for Research on Ageing, University of Southampton, Southampton, United Kingdom; 5 Department of Anthropology, Northwestern University, Evanston, IL; 6 Office of Population Studies Foundation, University of San Carlos, Cebu, Philippines; 7 Medical Research Council/Wits Developmental Pathways for Health Research Unit, Faculty of Health Sciences, University of the Witwatersrand, South Africa and Human Sciences Research Council, Johannesburg, South Africa; 8 Public Health Foundation of India, New Delhi, India; 9 Department of Pediatrics, Sitaram Bhartia Institute of Science and Research, New Delhi, India

**Keywords:** COHORTS, Consortium on Health Orientated Research in Transitional Societies, HAZ, Height-for-age z-scores, LMICs, Low- and middle-income countries, MC, Mid-childhood, MI, Multiple imputations, PR, Prevalence ratio, SES, Socioeconomic status

## Abstract

**Objective:**

To examine associations between maternal height and child growth during 4 developmental periods: intrauterine, birth to age 2 years, age 2 years to mid-childhood (MC), and MC to adulthood.

**Study design:**

Pooled analysis of maternal height and offspring growth using 7630 mother–child pairs from 5 birth cohorts (Brazil, Guatemala, India, the Philippines, and South Africa). We used conditional height measures that control for collinearity in height across periods. We estimated associations between maternal height and offspring growth using multivariate regression models adjusted for household income, child sex, birth order, and study site.

**Results:**

Maternal height was associated with birth weight and with both height and conditional height at each age examined. The strongest associations with conditional heights were for adulthood and 2 years of age. A 1-cm increase in maternal height predicted a 0.024 (95% CI: 0.021-0.028) SD increase in offspring birth weight, a 0.037 (95% CI: 0.033-0.040) SD increase in conditional height at 2 years, a 0.025 (95% CI: 0.021-0.029 SD increase in conditional height in MC, and a 0.044 (95% CI: 0.040-0.048) SD increase in conditional height in adulthood. Short mothers (<150.1 cm) were more likely to have a child who was stunted at 2 years (prevalence ratio = 3.20 (95% CI: 2.80-3.60) and as an adult (prevalence ratio = 4.74, (95% CI: 4.13-5.44). There was no evidence of heterogeneity by site or sex.

**Conclusion:**

Maternal height influences offspring linear growth over the growing period. These influences likely include genetic and non-genetic factors, including nutrition-related intergenerational influences on growth that prevent the attainment of genetic height potential in low- and middle-income countries.

Adult height is the cumulative result of the interaction between environment and genetics over the growing period. In developing countries, growth failure in the first 1000 days (conception to 2 years) of life^1^, ^2^, ^3^ is a strong determinant of final adult height.^2^ Among adults, short adult is associated with reduced human capital.^4^ Short maternal height is associated with low offspring birth size, childhood stunting, and reduced human capital,^5^, ^6^, ^7^, ^8^ likely in part due to maternal physical constraints on offspring growth in utero.^9^ Shorter women may have reduced protein and energy stores, smaller reproductive organ sizes, and limited room for fetal development.^10^, ^11^ These influence fetal growth via the placenta and infant growth through breast milk quantity and quality.^12^ Beyond this period, correlations between maternal and child heights are expected to be strongly influenced by genetics.^13^

Several studies have examined the cross-sectional association of maternal height with child size at birth and at selected postnatal ages,^6^, ^14^, ^15^ but there is less known about the relationship between maternal height and offspring growth over the life course. In particular, we are not aware of any studies examining the relationship between maternal height and offspring postnatal linear growth during specific, potentially critical, developmental periods. Such evidence would help inform policies and programs to prevent growth failure and to assess its intergenerational consequences. Estimating the separate impacts of maternal height on specific periods of growth is fraught with methodological difficulties because growth in distinct intervals is correlated within a child. We address this challenge by using growth modeling techniques that partition correlated longitudinal data into distinct and orthogonal components. We then relate maternal height to growth during these distinct periods. The objective of this study is to examine associations between maternal height and child growth during 4 developmental periods: intrauterine, birth to age 2 years, age 2 years to mid-childhood (MC), and MC to adulthood.

## Methods

We analyzed data from the 5 studies that participate in the Consortium on Health Orientated Research in Transitional Societies (COHORTS).^16^ These include the 1982 Pelotas Birth Cohort–Brazil,^17^ the Institute of Nutrition of Central America and Panama Nutrition Trial Cohort (INTCS)–Guatemala,^18^ the New Delhi Birth Cohort–India,^19^ the Cebu Longitudinal Health and Nutrition Survey (CLHNS)–Philippines,^20^ and the Birth to Twenty (BT20) Cohort–South Africa^21^ (Table I).

**Table I:** Descriptive characteristics of the 5 cohorts

Cohort	Design	Enrollment year	Cohort description	Analytic sample∗
Pelotas Birth Cohort, Brazil	Prospective cohort	1982	Children born in the city's maternity hospital (>99% of all births in 1982). All social classes included.	3535
INTCS, Guatemala	Community trial	1969-77	Intervention trial of a high-energy and protein supplement in women, and children <7 years in 1969 and born during 1969-1977 in 4 villages.	299
New Delhi Birth Cohort, India	Prospective cohort	1969-72	Babies born to an identified population of married women living in a defined area of Delhi. Primarily middle class sample.	836
CLHNS, Cebu, Philippines	Prospective cohort	1983-4	Pregnant women living in 33 randomly selected neighborhoods; 75% urban. All social classes included.	1892
BT20 Cohort, Johannesburg-Soweto, South Africa	Prospective cohort	1990	Babies born to pregnant women living in a defined urban geographical area in Johannesburg. Predominantly poor, Black sample.	375

*INTCS*, Institute of Nutrition of Central America and Panama Nutrition Trial Cohort; *CLHNS*, Cebu Longitudinal Health and Nutrition Survey; *BT20*, Birth to Twenty.

∗ Observations with data for maternal height and offspring birth weight, and height/length for data collections points of birth, 2 years, in MC, and in adulthood.

Maternal height and offspring anthropometric measurements were measured following site-specific protocols.^2^ Maternal height was measured using a stadiometer to the nearest 0.1 cm following standard procedures. All observers were trained in anthropometric techniques by experts and subsequently assessed for the reliability of their measurements, which fell within technical errors of measurement.^22^ Maternal height was measured at cohort enrollment/baseline in Brazil and the Philippines, and various time points around birth or in childhood for the other sites. Child data collected at birth, 2 years, a point we denote as MD, and adulthood were used in the present analysis. The MD point varied across sites because of variation in data collection schedules across the 5 cohorts. It was 48 months in Brazil, Guatemala, and India, 60 months in South Africa, and 102 months in the Philippines. We denote the interval between MC and adulthood as late childhood. Birth length was not available for Brazil and South Africa. Offspring birth weight was measured by the field research staff in Brazil, India, and Guatemala. In the Philippines, it was obtained from both field measurements and hospital records. In South Africa, it was obtained from birth records assessed for reliability.^23^ Offspring attained height-for-age z-scores (HAZ) at age 2, at MC, and in adulthood were calculated using the 2006/7 World Health Organization standard reference curves.^24^, ^25^ To compute the adult HAZ we used the tabulated Lambda, Mu, Sigma (the child growth modeling method/parameters for the World Health Organization curves) values for age 19 years, under the assumption that adult height is substantively attained by this age.

### Statistical Analyses

We included in the analysis all mother–child pairs with available anthropometry data in all periods (n = 7630). One or more predictors were missing for 19% of the analytic sample. We assumed the missing predictors (maternal height and household wealth) to be missing at random, and generated 5 multiple imputations (MI) using imputation chain equations. Combined estimates were obtained using MI inference rules.^26^ Analyses conducted with the available non-missing data (by list-wise deletion) had similar results but with wider CIs and, consequently, we kept the results of the MI analyses.

Maternal height was the primary exposure. Child growth (birth weight, height changes during the early, middle, and late childhood periods) and attained height (all in z-scores) were the outcome measures.

To address the challenge of collinearity of growth measures, we computed conditional height, the child-specific residual obtained from linear site-specific regression of height at each age on birth weight and all prior height measurements.^27^ Conditional height represents the change in height within a growth period relative to the child's prior height, in the context of the mean growth pattern of the population. Conditional variables are uncorrelated with each other. We used birth weight as the anchor for all conditional heights.

We defined offspring childhood stunting at 2 years as HAZ values <−2 SD.^24^ Using the same threshold of −2 SD, short stature in adulthood was defined as height <150.1 cm among females and <161.9 cm among males.^25^ We used study-specific measures of socioeconomic status (SES) at offspring birth, categorized into quintiles (1 = poorest) as a measure of SES during childhood. The SES quintiles are site-specific, and were estimated from maternal education and paternal occupation for India, and by asset scores for all other study sites.

Descriptive characteristics are presented as means (SD) for continuous variables or as percentages for categorical variables. We assessed the secular trends in adult heights across the 2 generations by subtracting maternal height from offspring adult height. We used correlation coefficients and generalized linear regression models to determine associations between maternal height and offspring growth measures. Similarly, we used generalized estimating equations models with robust error variances to estimate the prevalence ratio (PR) of offspring stunting at 2 years and adulthood using maternal height as the main predictor. Furthermore, we assessed whether the association of maternal and offspring adult shortness was mediated through childhood stunting at age 2 years. To determine this, we developed separate multivariate estimating equation models using maternal shortness or childhood stunting as predictors, followed by a model that included both predictors. We adjusted for household wealth (SES), study site, offspring sex, and birth order (categorized as firstborns and non-firstborns), based on documented associations of these variables with child growth.^6^, ^28^ We assessed whether relationships for the Philippines, where height was measured at 102 months, differed from those found for other cohorts, where height was measured at 48 or 60 months. The Guatemala cohort comes from a nutrition trial of Atole (a high-nutrient density supplement) versus Fresco (INCAPARINA, Guatemala City, Guatemala).^29^ We found significant differences in childhood heights and conditional height among the trial arms. Consequently, we created a nutrition intervention variable (3 categories: 1 = Atole, 2 = Fresco [for the 2 Guatemala intervention arms], and 3 = no intervention category, for all the other study sites) and adjusted for it in all pooled regression analyses.

We tested for heterogeneity of associations by site and offspring sex by examining site- and sex-specific estimates and with a formal statistical test of heterogeneity. Because no differences were observed by inspection, and no heterogeneity was detected at *P* < .05, we conducted pooled analyses with adjustment for sex and site. Standard regression diagnostic procedures were followed for all models. All analyses were conducted with PC-SAS 9.3 (SAS, Cary, North Carolina). Statistical significance was set at a 2-tailed *P* value of <.05.

## Results

Mean maternal age at offspring birth was 26.4 years. Maternal height varied across sites (range 148.6 ± 4.8 cm in Guatemala to 158.3 ± 6.7 cm in South Africa). Mean birth weight ranged from 2.8 ± 0.4 kg (India) to 3.2 ± 0.5 Kg (Brazil). Stunting prevalence at 2 years was highest in Guatemala (84%) and lowest in Brazil (12%). Offspring adults were shortest in Guatemala and the Philippines and tallest in Brazil and South Africa (Table II). Offspring women were 2.3 (95% CI: 1.7-2.9) cm and men were 15.0 cm (95% CI: 14.4-15.6) taller than their mothers (Table III).

**Table II:** Characteristics of mothers and their offspring, by cohort (overall n = 7630)

Variables	Brazil, n = 3588	Guatemala, n = 301	India, n = 1326	Philippines, n = 1892	South Africa, n = 523
	Mean (SD)/%	Mean (SD)/%	Mean (SD)/%	Mean (SD)/%	Mean (SD)/%
Mother					
Height, cm	156.5 (6.0)	148.6 (4.8)	152.7 (5.4)	150.6 (4.9)	158.3 (6.7)
Short stature (%)∗	10.9	57.1	34.9	42.7	7.8
Age at delivery, y	26.3 (6.2)	27.7 (7.1)	26.7 (5.7)	26.3 (6.1)	25.4 (6.2)
Offspring					
Sex, male (%)	52.4	52.2	53.1	53.2	50.5
Birth weight, kg	3.2 (0.5)	3.1 (0.5)	2.8 (0.4)	3.0 (0.4)	3.1 (0.5)
Birth weight (z-score)	−0.18 (1.0)	−0.50 (1.0)	−1.1 (1.0)	−0.66 (1.0)	−0.47 (1.0)
Birth length, cm	NA	49.3 (2.3)	48.3 (2.1)	49.1 (2.0)	NA
Length at 2 y, cm	80.7 (4.9)	77.4 (3.6)	80.4 (3.6)	79.3 (3.6)	83.0 (3.9)
Stunted at 2 y (%)	12.0	84.1	47.0	67.3	24.9
MC height, cm	97.5 (5.1)	92.8 (3.6)	94.8 (4.2)	117.7 (5.5)	108.0 (4.6)
Adult height, males, cm	173.8 (6.9)	163.0 (6.1)	169.8 (6.2)	163.0 (5.9)	171.2 (6.6)
Adult height, females, cm	160.9 (6.2)	151.1 (5.2)	154.9 (5.6)	151.1 (5.4)	159.5 (6.1)
Short adult, males (%)†	3.5	44.0	9.5	41.0	8.0
Short adult, females (%)	3.4	43.1	19.1	42.3	6.2

*NA*, not available.

∗ Short mother: maternal height <150.1 cm.

† Short: adult height z-scores <−2 SD at 19 years. Total sample size (n = 7630) includes imputed values.

**Table III:** Intergenerational mean (95% CI) differences in adult height, cm between mother–child pairs∗

Analyses	Mean differences (95% CI) (child adult height-maternal height), cm	
	Male child	Female child
Pooled	15.0 (14.4-15.6)	2.3 (1.7-2.9)
Brazil	17.1 (17.4-16.8)	4.6 (4.3-4.9)
Guatemala	14.6 (13.7-15.5)	2.4 (1.6-3.3)
India	17.9 (17.4-18.4)	2.9 (2.3-3.4)
Philippines	12.4 (12.0-12.7)	0.6 (0.2-0.9)
South Africa	13.3 (12.2-14.1)	1.1 (0.2-2.0)

∗ All estimates obtained from 5 multiple imputation datasets.

### Associations between Maternal Height and Child Growth Measures

The correlations between maternal height and offspring length/height measures at various ages ranged from 0.15-0.55 (all *P* < .001; Table IV). Correlations between maternal height and birth measures were weaker (birth weight r = 0.19, birth length r = 0.15) compared with later periods (r = 0.42, 0.47, and 0.54 for height at 2 years of age, MC, and adulthood, respectively). In models that used the offspring attained height measures, a 1-cm mean increase in maternal height was associated with 0.024 (95% CI: 0.021-0.028) SD increase in offspring birth weight z-score and with 0.078 (95% CI: 0.074-0.083), 0.080 (95% CI: 0.077-0.085), and 0.082 (95% CI: 0.079-0.086) SD increases in attained HAZ at 2 years, MC, and in adulthood, respectively. In models that used conditional offspring height measures, a 1-cm increase in maternal height was associated with 0.037 (95% CI: 0.033-0.040), 0.025 (95% CI: 0.021-0.029), and 0.044 (95% CI: 0.040-0.048) SD unit increases in offspring conditional height at 2 years, MC, and adulthood, respectively.

**Table IV:** Associations between maternal height (cm) and offspring growth measures for 5 cohorts (n = 7630)

Child growth measures	Partial correlations (r) (pooled all sites)∗	Linear associations with maternal height, cm
		Pooled analyses (all sites)
Size at birth, z-scores:		**Estimate, β (95% CI)**†
Birth weight	0.19	0.024 (0.021-0.028)
Birth length‡	0.15	0.034 (0.026-0.042)
Attained size, z-scores:		
Height at 2	0.42	0.078 (0.074-0.083)
Height at MC§	0.47	0.080 (0.077-0.085)
Adult height	0.54	0.082 (0.079-0.086)
Conditional growth, z-scores:		
Conditional height 0-2 y	0.24	0.037 (0.033-0.040)
Conditional height 2-MC	0.15	0.025 (0.021-0.029)
Conditional height MC-adulthood	0.26	0.044 (0.040-0.048)

∗ Pearson partial correlations adjusted for offspring sex, site, and SES.

† Regression coefficients calculated from multiply imputed analyses.^27^

‡ Pooled estimates excluding Brazil and South Africa. All *P* values for partial correlation coefficients were significant at *P* < .001.

§ MC: 48 months of age for Brazil, Guatemala, and India, 60 months of age for South Africa, and 102 months of age for Philippines. Mother–child correlation coefficients for attained height, cm are 0.32, 0.15, and 0.54 at 2 years, MC, and adulthood, respectively. All models adjusted for offspring sex, site, and SES (household wealth in quintiles), child birth order, and nutrition supplementation. Mother–child adjusted linear associations for attained adult height cm β = 0.568 (95% CI: 0.546-0.590).

### Maternal Height and Offspring Stunting

Maternal height was inversely associated with the prevalence of offspring stunting at 2 years (PR = 0.88 [95% CI: 0.87-0. 89]; Table V). Compared with taller mothers, short mothers were more likely to have a child who was stunted at 2 years (PR = 3.20 [95% CI: 2.80-3.60]) and as an adult (PR = 4.74 [95% CI: 4.13-5.44]). Childhood stunting was strongly associated with subsequent adult shortness (PR = 12.81; 95% CI: 10.70-15.35). Simultaneous modeling of both maternal short stature and offspring stunting at age 2 years attenuated these associations only modestly (Table V). We controlled for nutrition intervention in all linear regression models considered (pooled analyses); however, associations between maternal height and child growth measures were unaffected by this variable. Results of analysis differentiating the Philippines from the other 4 cohorts indicated similar association patterns.

**Table V:** PRs (95% CI) for offspring stunting and maternal height in 5 cohorts (n = 7630)

Models	Adjusted PR∗	
	Childhood stunting (at 2 y) PR (95% CI)	Stunted adult† PR (95% CI)
Independent effects models		
Maternal height, cm	0.88 (0.87-0.89)	0.83 (0.82-0.84)
Short mother (<150.1 cm)	3.20 (2.80-3.58)	4.74 (4.13-5.44)
Offspring stunting at 2 y	-	12.81 (10.70-15.35)
Joint effect models‡		
Short Mother	-	3.38 (2. 91-3.92)
Offspring Stunting at 2 y	-	10.34 (8.61-12.43)

∗ Estimates were calculated from 5 multiple data sets.^26^ All models have been adjusted for child sex, site + SES (household wealth), childbirth order, and nutrition supplementation. Site–sex interaction terms were tested for heterogeneity, found nonsignificant, and dropped from the final models.

† Stunted adult: Adult height <150.1 cm and <161.9 cm for female and male offspring, respectively.

‡ Models include both short mother and offspring stunting at 2 years.

## Discussion

We examined association between maternal height and offspring birth weight and height changes during 4 periods of childhood using data from birth cohorts in 5 low- and middle-income countries (LMICs). Despite marked difference in anthropometric characteristics (eg, stunting, adult height) across the cohorts, we observed consistent associations across the 5 cohorts as well as by sex. Maternal height was positively associated with all offspring height measures. Consistent with previous studies,^5^, ^6^, ^7^ our analysis found maternal height to be associated with birth size. Our study moves beyond earlier studies of the association between maternal height and size at various points in childhood,^6^, ^30^, ^31^ to consider associations with linear growth using conditional measures of growth that are uncorrelated across ages over the entire growing period.

Maternal height was significantly associated with offspring birth weight, and with all postnatal attained and conditional height measures. The observed correlations and linear associations were consistent with studies from high-income country settings for birth size and adult height.^15^, ^31^, ^32^, ^33^ The associations of maternal height with offspring birth weight were weaker than associations with conditional height at 2 years and at adulthood, which represents growth from MC to adulthood. Given the context of the cohorts, all from LMICs, we expected that the associations with early growth would have been the strongest, reflecting the biological role of the maternal milieu during pregnancy and lactation, in addition to genetics. Growth prior to age 2 years is influenced by environmental factors such as maternal nutrition, feeding practices, dietary quality and quantity, and infections, which may obscure genetic influences.^12^ Perhaps the less than expected relationship between maternal height and early growth may also be due to our inability to properly quantify genetic influences and less canalized growth during this period. Growth from MC to adulthood is a period of low infection and where linear growth in these cohorts is similar to that observed in the World Health Organization reference population, reflecting the achievement of growth potential.^1^, ^2^ Thus, growth from MC to adulthood may reflect largely genetic influences. Unlike the conditional height measures, associations between maternal and offspring attained heights were identical at offspring ages 2 years, MC, and adulthood. This is likely due to the strong inter-correlations (0.24-0.63, in our study data) in heights across ages, emphasizing the advantages of using conditional growth variables^27^ for our research question. Because the MC point was assessed at 102 months (8.5 years) of age in the Philippines, we considered whether the results might be influenced by early onset of puberty. This seems unlikely. For example, in an examination of maturation patterns in the Philippines cohort, early maturing girls were defined as those whose age at menarche was below 1 SD of the mean of the average maturing girls' in the entire population.^34^ The mean and SD for early maturing girls was 13.6 ± 1.5 years and the cutoff point for 2 SDs below the mean of this subpopulation corresponds to 10.6 years. It is therefore likely that for the vast majority of children, the data point at 8.5 years in the Philippines cohort represents a prepubertal height measure. The inclusion of a small number of potential outliers with precocious puberty would not cause any substantive bias in our estimates. Indeed, the pattern of relationships observed in the data for the Philippines cohort was similar to that observed in the 4 other cohorts (data not shown).

Short maternal stature has been associated with stunting in children.^35^ Our study confirms this association and extends it through adulthood. Short mothers (<150.1 cm) were 3.2 times more likely to have a stunted child at 2 years compared with taller mothers; this is consistent with previous studies from other LMICs.^14^, ^35^ The protective influence of maternal height against offspring stunting agrees with prior observations that tall mothers have increased reproductive success (fertility, child survival) in stressed environments.^36^, ^37^ We use stunting and short adult stature in their statistical sense as representing the lower end of the distribution of stature. It is also noted that there are multiple reasons for short adult stature in various populations, with stunting merely being an indicator of deprivation in the first 1000 days.

As adults, offspring in our cohorts were on average taller than their mothers. These secular increases in height are probably due to improved economic, nutrition, hygiene, and other factors in recent decades in LMICs. The secular trends varied across the 5 cohorts, probably related to the timing and success of investments in maternal/child health and nutrition. Although growth is known to exhibit sexual dimorphism, we found no significant heterogeneity of associations by offspring sex across the 5 study sites.

In poor settings, the height of a mother is a proxy for nutrition during her own growth and development.^38^ Short women in the LMICs are also likely to be poorer and live in more restricted circumstances, so complementary foods are also likely to be less nutritious. To break this intergenerational cycle, interventions to prevent stunting yield maximum benefits when applied within the first 1000 days of life: pregnancy and the first 2 years.^3^, ^12^ Exposure to the Guatemala nutrition trial during the first 3 years of life in girls impacted on their own children's size at birth and height in childhood,^29^ highlighting the long-term returns for nutritional interventions targeted at critical developmental periods of maternal childhood growth. Public health programs aimed at preventing malnutrition and childhood stunting is, therefore, an intergenerational investment in human capital, and likely to operate through alleviation of maternal childhood growth retardation, and increasing adult stature.

The strengths of this study include the use of cohort data with growth measures from birth into adulthood, large sample sizes from 5 different countries, and the use of appropriate statistical methods to account for otherwise-correlated measures of growth. The use of MI techniques ensured efficient use of the available data and improved precision around estimates. Birth length data was not available for Brazil and South Africa; we used birth weight to address this. In our data, birth weight and birth length is correlated at 0.7; hence we do not believe that this introduced bias into our analyses. Further, we repeated the analyses using either a birth length anchor or birth weight anchor for the 3 sites for which we have both measures, but the results were very similar. Additionally, data on paternal height was not available. Because of this limitation, we were not able to distinguish the intergenerational importance of paternal or mid-parental height for offspring growth.

## Appendix 1

The writing group was responsible for the research construct and methods development for this paper. Authors included at least 2 individuals from each study site, and had major roles in the collection of data contributed to the COHORTS collaboration. The members of the COHORTS Group include: Universidade Federal de Pelotas, Brazil: Cesar G. Victora, MD, PhD, Fernando C. Barros, PhD, Denise Gigante, PhD, Pedro C. Hallal, PhD, and Bernardo L. Horta, PhD; Hubert Department of Global Health, Rollins School of Public Health, Emory University, Atlanta, Georgia: Reynaldo Martorell, PhD, Aryeh Stein, PhD, O.Yaw Addo, MS, PhD, and Wei Hao, MA; Instituto de Nutrición de Centra América y Panamá, Guatemala City, Guatemala: Manual Ramirez-Zea, MD, PhD; Sunderlal Jain Hospital, New Delhi, India: Santosh K. Bhargava, MD; Sitaram Bhartia Institute of Science and Research, New Delhi, India: Harshpal Singh Sachdev, MD; Public Health Foundation of India, New Delhi, India: Poornima Prabhakaran, MBBS, MSc; Medical Research Counci Lifecourse Epidemiology Unit, University of Southampton, Southampton, United Kingdom: Clive Osmond, PhD and Caroline Fall, DM; Department of Nutrition, University of North Carolina at Chapel Hill, Chapel Hill, North Carolina: Linda Adair, PhD; Office of Population Studies, University of San Carlos, Cebu City, Philippines: Isabelita Bas, MA, Nanette Lee, PhD, and Judith Borja, PhD; University of Leeds, Leeds, United Kingdom: Darren Dahly, PhD; Department of Anthropology, Northwestern University, Evanston, Illinois: Christopher Kuzawa, PhD; Developmental Pathways for Health Research Unit, Department of Pediatrics, University of the Witwatersrand, Johannesburg, South Africa: Linda Richter, PhD, Shane Norris, PhD, and Julia De Kadt, PhD; Department of Psychiatry, University of Oxford, Oxford, United Kingdom: Alan Stein, FRCPsych.

## Appendix 2

The current COHORTS analysis is supported by the Bill and Melinda Gates Foundation and The Wellcome Trust (United Kingdom). Funding for data collection for the five Cohorts were: Institute of Nutrition of Central America, Panama Nutrition Trial Cohort (Guatemala), US National Institutes of Health, and US National Science Foundation; Pelotas Birth Cohort (Brazil) Wellcome Trust; New Delhi Birth Cohort Study (India), Indian Council of Medical Research, US National Center for Health Statistics, Medical Research Council (United Kingdom), and British Heart Foundation; Birth to Twenty Cohort (South Africa), Wellcome Trust, Human Sciences Research Council, South African Medical Research Council, South-African Netherlands Program on Alternative Development, Anglo-American Chairman's Fund and University of the Witwatersrand; and Cebu Longitudinal Health, and Nutrition Survey (the Philippines) US National Institutes of Health. The authors declare no conflicts of interest.
